# Air sampling and analysis method to determine worker exposure levels to *N*-isopropyl-*N*′-phenyl-*p*-phenylenediamine

**DOI:** 10.1093/joccuh/uiaf059

**Published:** 2025-10-22

**Authors:** Tomiko Tashiro, Akito Takeuchi, Kenta Ishii, Ai Yamada, Osamu Nishinoiri, Ginji Endo, Mariko Ono-Ogasawara

**Affiliations:** Kinki Osaka Safety and Health Service Center, Japan Industrial Safety and Health Association, 2-3-8 Tosabori, Nishi-Ku, Osaka 550-0001, Japan; Kinki Osaka Safety and Health Service Center, Japan Industrial Safety and Health Association, 2-3-8 Tosabori, Nishi-Ku, Osaka 550-0001, Japan; Occupational Health Research and Development Center, Japan Industrial Safety and Health Association, 5-35-2 Shiba, Minato-Ku, Tokyo 108-0014, Japan; Kanto Regional Safety and Health Service Center, Japan Industrial Safety and Health Association, 1st fl, Sigma Bldg, 3-7-12 Shibaura, Minato-ku, Tokyo 108-0023, Japan; Laboratory of Environmental Toxicology and Carcinogenesis, School of Pharmacy, Nihon University, 7-7-1 Narashinodai, Funabashi, Chiba 274-8555, Japan; Kinki Osaka Safety and Health Service Center, Japan Industrial Safety and Health Association, 2-3-8 Tosabori, Nishi-Ku, Osaka 550-0001, Japan; Department of Pathophysiological Laboratory Sciences, Nagoya University Graduate School of Medicine, 1-1-20 Daiko-minami, Higashi-Ku, Nagoya 461-8673, Japan; Kanto Regional Safety and Health Service Center, Japan Industrial Safety and Health Association, 1st fl, Sigma Bldg, 3-7-12 Shibaura, Minato-ku, Tokyo 108-0023, Japan; Kinki Osaka Safety and Health Service Center, Japan Industrial Safety and Health Association, 2-3-8 Tosabori, Nishi-Ku, Osaka 550-0001, Japan; National Institute of Occupational Safety and Health, Nagao 6-21-1, Tama-Ku, Kawasaki 214-8585, Japan

**Keywords:** air sampling method, high-performance liquid chromatography, *N*-isopropyl-*N*′-phenyl-*p*-phenylenediamine, personal exposure measurement, workplace air

## Abstract

**Objectives**: This study aimed to develop a method for determining workers’ exposure concentrations to airborne *N*-isopropyl-*N*′-phenyl-*p*-phenylenediamine (IPPD).

**Methods**: A hydrophobic polytetrafluoroethylene (HPB PTFE) membrane filter was used as the sampling medium. IPPD was extracted from the HPB PTFE filter with acetonitrile, and its concentration in the extracted solution was determined using a high-performance liquid chromatograph equipped with a photodiode array detector. The validating method was performed using the following parameters: extraction and retention efficiency, storage stability, method quantitation limit, and reproducibility.

**Results**: The IPPD extraction efficiency from the spiked HPB PTFE filters was 96%-100%. The IPPD retention efficiencies on the HPB PTFE filters were 72%-99%, with relative standard deviations indicating the overall reproducibility of 0.9%-3.1%. IPPD on the HPB PTFE filter remained stable for at least 7 days at 4°C. The method quantitation limit was 12.5 μg/sample.

**Conclusions**: We successfully developed a method to measure workers’ exposure to airborne IPPD within the concentration range of 0.05-4 mg/m^3^, which will assist risk assessments.

## Teaser

Key points

**What is already known on this topic:**  *N*-isopropyl-*N*′-phenyl-*p*-phenylenediamine (IPPD) is mainly used as an anti-aging agent in the rubber industry. Owing to its harmfulness, an occupational exposure limit for IPPD was proposed by the Deutsche Forschungsgemeinschaft (DFG, German Research Foundation). However, no method exists to measure worker exposure concentrations to airborne IPPD.

**What this study adds:** This research introduces a method that uses a hydrophobic polytetrafluoroethylene membrane filter as a sampling medium. This method enables the measurement of personal exposure concentrations of IPPD ranging from 0.05 to 4 mg/m^3^ in a 4-hour sampling period.

**How this study might affect research, practice, or policy:** The developed method provides a means to estimate IPPD exposure levels among workers, and then will aid in preventing their exposure to IPPD.

## 1. Introduction

*N*-isopropyl-*N*′-phenyl-*p*-phenylenediamine (IPPD, CAS RN, 101-72-4), a solid with a very low vapor pressure at ambient temperature,^1^^,^^2^ is used mainly as an anti-aging agent in the rubber industry.^2^ The Ministry of Health, Labour, and Welfare (MHLW) of Japan selected IPPD as a target chemical in a project on the risk assessment of chemicals in the workplace from 2019 to 2021. Although the Japan Society for Occupational Health and the American Conference of Governmental Industrial Hygienists have yet to propose an occupational exposure limit for IPPD, the Deutsche Forschungsgemeinschaft (DFG, German Research Foundation)^2^ proposed 2 mg/m^3^ as the inhalable fraction (Maximale Arbeitsplatzkonzentrationen [maximum workplace concentrations], MAK). However, to the best of our knowledge, no methods have been reported to measure worker exposure concentrations to airborne IPPD; therefore, the purpose of this study was to establish such a method.

## 2. Materials and methods

### 2.1. Materials

IPPD was purchased from Tokyo Chemical Industry. High-performance liquid chromatography (HPLC)-grade acetonitrile and methanol, and guaranteed reagent-grade ammonium acetate were obtained from Kanto Chemical Co, Inc. Ultra-pure water was obtained from a PURELAB flex-3 (Organo Corp). Ammonium acetate buffer (20 mM, pH 6.6) was prepared by dissolving ammonium acetate in ultra-pure water. A standard stock solution of IPPD was prepared in acetonitrile and stored at 4°C. A glass-fiber (GF) filter (GB-100R; 0.6-μm pore size, 37-mm diameter, Catalog No. 36321037), hydrophilic polytetrafluoroethylene (HPL PTFE) membrane filter (H050A025A; 0.50-μm pore size, 25-mm diameter, Catalog No. 13050802), and hydrophobic polytetrafluoroethylene (HPB PTFE) membrane filter (T050A025A; 0.50-μm pore size, 25-mm diameter, Catalog No. 12050002) were purchased from Advantec Toyo Kaisha, Ltd. A nylon (NY) membrane filter (0.45-μm pore size, 47-mm diameter, Catalog No. 1213825), polypropylene (PP) membrane filter (0.45-μm pore size, 25-mm diameter, Catalog No. 1212379), and HPB PTFE membrane filter (0.45-μm pore size, 25-mm diameter, Catalog No. 1215492) were purchased from GVS S.p.A. A polyvinyl chloride (PVC) membrane filter (ACCU-CAP; 5.0-μm pore size, 37-mm diameter, Catalog No. 225-8516GLA) and an HPB PTFE membrane filter (0.5-μm pore size, 25-mm diameter, Catalog No. 225-3708) were purchased from SKC Inc. A quartz fiber (QF) filter (2500QAT-UP; 47-mm diameter) was purchased from Pall Corp. The 37-mm and 47-mm diameter filters (GF, PVC, and QF) were used after punching them to 25 mm in diameter.

### 2.2. Method validation procedure

The proposed method was validated for the following parameters, following the MHLW guidelines^3^: extraction efficiency, retention efficiency, stability of samples in storage, method limit of quantitation, and reproducibility. The procedures for testing extraction efficiency, retention efficiency, and storage stability were as follows: IPPD-spiked filters were prepared by spiking 20-μL aliquots of various concentrations of IPPD standard solutions onto the sampling filters and drying thoroughly at room temperature. For the extraction efficiency tests, IPPD-spiked filters were used without further treatment. For the retention efficiency and storage stability tests, IPPD-spiked filter were set into filter cassettes in the aerosol sampling head of an inhalable fraction and vapor (IFV) Pro Sampler (Catalog No. 225-49, SKC Inc), and then room air (temperature, 21.4°C-24.4°C; relative humidity, 25%-27%) was drawn through it using an SKC Air Check 2000 (Catalog No. 210-2002, SKC Inc) sampling pump set at a flow rate of 1 L/min for 4 hours (which is the minimum sampling time required by the MHLW guidelines^3^). For the storage stability test, after drawing room air, the samplers were sealed without removing the filters from the cassette and stored in a refrigerator (4°C) for 7 days. The amounts of IPPD spiked onto the sampling filters ranged from 5.00 to 1000 μg (7 different amounts) for the extraction and retention efficiency tests, and from 12.5 to 1000 μg (4 different amounts) for the storage stability test. These amounts corresponded to approximately 0.02-4 mg/m^3^ and 0.05-4 mg/m^3^, representing approximately 1/100 to 2 times the MAK value and 1/40 to 2 times the MAK value proposed by the DFG. To obtain a calibration curve, the IPPD standard solutions were prepared from the standard stock solution by dilution with acetonitrile at 9 concentrations, namely, 1.00, 2.50, 5.00, 7.50, 10.0, 50.0, 100, 150, and 200 μg/mL, and then analyzed. Calibration curves were constructed by plotting the peak area of IPPD versus its respective concentrations.

### 2.3. Analytical procedure and instrumental conditions

The sampled filter was removed from the filter cassette in the aerosol-sampling head of the IFV Pro Sampler and placed in a glass test tube. Acetonitrile (5 mL) was added to the tube, which was vigorously shaken using an MTV-100 multitube vortexer (Hangzhou Allsheng Instruments Co, Ltd) for 5 minutes. The extracted solution was filtered through a DISMIC-13HP020AN filter (Advantec Toyo Kaisha, Ltd) and placed in an autosampler vial for analysis. HPLC analysis was performed using a Prominence UFLC system with an SPD-M20A photodiode array (PDA) detector (Shimadzu Corporation). Separation was achieved under the following conditions: column, InertSustainSwift C18 (150 × 3.0 mm ID, 5 μm; GL Sciences, Inc); flow rate, 0.5 mL/min; temperature, 40°C; mobile phase, 20 mM ammonium acetate buffer (pH 6.6):acetonitrile (55:45, v/v); elution mode, isocratic elution; PDA acquisition wavelength, 190-600 nm; detection wavelength, 287 nm; injection volume, 5 μL.

## 3. Results

To select a suitable filter for sampling IPPD, 9 different types (PP, PVC, GF, HPL PTFE, QF, NY, HPB PTFE GVS, HPB PTFE SKC, and HPB PTFE Advantec filter) were evaluated by performing extraction efficiency and retention efficiency tests (test conditions: spiked amount, 50.0 μg; sampling volume, 240 L; room air temperature and relative humidity, 23.4°C-24.4°C and 25%-28%; *n* = 5, respectively). When evaluating the NY and PVC filters, methanol was used instead of acetonitrile as the diluent for the standard solution used for spiking IPPD into the filters and as the extraction solution. Although all filters showed good extraction efficiencies (mean ± SD; 96 ± 1.4%, 95 ± 2.3%, 99 ± 0.5%, 99 ± 1.5%, 100 ± 1.7%, 101 ± 2.7%, 99 ± 2.5%, 100 ± 0.6%, and 100 ± 1.8% for PP, PVC, GF, HPL PTFE, QF, NY, HPB PTFE GVS, HPB PTFE SKC, and HPB PTFE Advantec filters, respectively), their retention efficiencies varied widely (mean ± SD; 45 ± 1.6%, 59 ± 2.9%, 68 ± 4.0%, 71 ± 6.4%, 72 ± 1.8%, 72 ± 3.2%, 94 ± 1.2%, 94 ± 1.2%, and 96 ± 1.1% for PP, PVC, GF, HPL PTFE, QF, NY, HPB PTFE GVS, HPB PTFE SKC, and HPB PTFE Advantec filters, respectively).

**Table 1:** Extraction efficiency, retention efficiency, and storage stability tests.^a^

	**Extraction efficiency**				**Retention efficiency**				**Storage rate (%, *n* = 5)**																			
	**(%, *n* = 5)**				**(%, *n* = 5)**				**0 days**				**1 day**				**3 days**				**5 days**				**7 days**			
**Spiked amount, μg**	**Mean**	**±**	**SD**	**RSD**	**Mean**	**±**	**SD**	**RSD**	**Mean**	**±**	**SD**	**RSD**	**Mean**	**±**	**SD**	**RSD**	**Mean**	**±**	**SD**	**RSD**	**Mean**	**±**	**SD**	**RSD**	**Mean**	**±**	**SD**	**RSD**
5.00	96	±	1.4	1.4	72	±	2.2	3.1	—	±	—	—	—	±	—	—	—	±	—	—	—	±	—	—	—	±	—	—
10.0	99	±	0.8	0.8	87	±	1.3	1.5	—	±	—	—	—	±	—	—	—	±	—	—	—	±	—	—	—	±	—	—
12.5	99	±	0.9	0.9	95	±	1.9	2.0	100	±	1.0	1.0	94	±	0.9	1.0	94	±	1.2	1.3	97	±	1.5	1.5	91	±	1.6	1.8
25.0	100	±	0.5	0.5	97	±	2.1	2.1	—	±	—	—	—	±	—	—	—	±	—	—	—	±	—	—	—	±	—	—
50.0	100	±	1.8	1.8	96	±	1.1	1.1	100	±	0.8	0.8	97	±	0.5	0.5	97	±	0.8	0.8	98	±	1.1	1.1	96	±	0.4	0.4
500	99	±	1.5	1.5	99	±	0.9	0.9	100	±	1.9	2.0	100	±	0.8	0.8	99	±	1.2	1.3	98	±	1.2	1.3	97	±	1.0	1.0
1000	99	±	0.4	0.4	98	±	1.3	1.3	100	±	1.1	1.1	99	±	0.4	0.4	100	±	1.1	1.1	98	±	0.4	0.4	99	±	1.0	1.0

Abbreviations: HPB PTFE, hydrophobic polytetrafluoroethylene; IFV, inhalable fraction and vapor; IPPD, *N*-isopropyl-*N*′-phenyl-*p*-phenylenediamine; RSD, relative standard deviation.

a The IPPD-spiked HPB PTFE Advantec filter was set into the filter cassette in the aerosol sampling head of the IFV Pro Sampler, and then room air (temperature, 21.4°C-24.4°C; relative humidity, 25%-27%) was drawn through it using the sampling pump at flow rate of 1 L/min for 4 hours. For the storage stability test, the samplers after drawing room air were sealed without removing the filters from the filter cassette and then stored in a refrigerator (4°C) for 7 days. The spiked amounts correspond to air concentrations of approximately 0.02-4 mg/m^3^.

**Figure 1:**
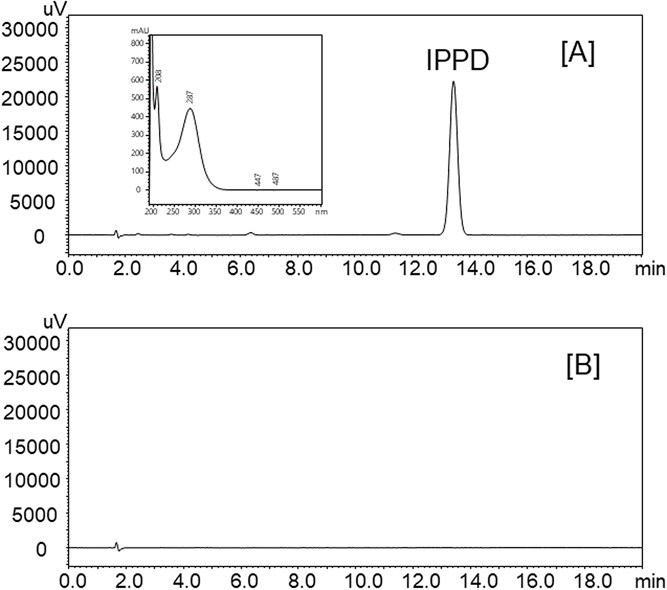
Chromatograms of solutions extracted from the hydrophobic polytetrafluoroethylene (HPB PTFE) Advantec filter after 240 L of room air was passed through it with (A) and without (B) adding 50.0 μg of *N*-isopropyl-*N*′-phenyl-*p*-phenylenediamine (IPPD).

The validation results of the developed method using the HPB PTFE Advantec filter are as follows. The extraction and retention efficiencies were 96%-100% and 72%-99%, respectively (Table 1). Relative standard deviations (RSDs), representing the overall reproducibility of the proposed method, were calculated from the results of the retention efficiency tests and ranged from 0.9% to 3.1% (Table 1). The storage rates were calculated by comparing the amount of IPPD remaining on the HPB PTFE Advantec filters after storage with those analyzed before storage. After 7 days of storage in a refrigerator (4°C), the storage rate was greater than 91% (Table 1). The linearity of the calibration curve was within the range of 1.00-200 μg/mL, with a coefficient of determination (*R*^2^) greater than 0.999. The instrumental limit of quantitation, calculated as 10 times the SD (*n* = 5) of the peak area of the lowest standard solution (1.00 μg/mL) divided by the slope of the calibration curve and multiplied by the volume of the extraction solution (5 mL), was 0.300 μg/sample. The method limit of quantitation, defined as the smallest spiked amount of IPPD that yielded greater than 90% retention efficiency within the range of the retention efficiency test, was 12.5 μg/sample. Under the established analytical conditions, the absorption spectrum of the IPPD peak showed maximum absorption at approximately 287 nm (Figure 1A, inset), and was therefore used as the detection wavelength. Chromatograms of the solutions extracted from the HPB PTFE Advantec filter after 240 L of room air was passed through it with and without the addition of IPPD are shown in Figure 1A and B, respectively. IPPD was not detected in Figure 1B.

## 4. Discussion

To our best knowledge there have been no reports of determination methods for airborne IPPD, including workplace air, although several methods have been reported for the determination of IPPD in other matrices (urine and rubber boots) using HPLC ultraviolet detection (UV),^4^^-^^7^ which we referenced when developing the method reported here. IPPD is expected to exist as an aerosol in workplace air because it is a solid with a very low vapor pressure at ambient temperature.^1^^,^^2^ Therefore, we adopted a filter-sampling method. Comparison of extraction and retention efficiencies for 9 different types of filters suggested that the HPB PTFE filters are suitable for sampling IPPD, because the retention efficiencies of all 3 types exceeded 94%. Therefore, we adopted the HPB PTFE Advantec filter, which showed the highest retention efficiency among the 3 HPB PTFE filters (Advantec vs GVS, *P* = .0159; Advantec vs SKC, *P* = .0308; GVS vs SKC, *P* = .7271; Student *t* test). However, when using the HPB PTFE Advantec filter, special attention should be paid to not exceed a maximum sampling time of 4 hours, because our results that the retention efficiency was lower than the extraction efficiency for each spiked amount (extraction efficiency vs retention efficiency, *P* < .0001 for 5.00 and 10.0 μg, *P* = .0050 for 12.5 μg, *P* = .0175 for 25.0 μg, *P* = .0022 for 50.0 μg, *P* = .5821 for 500 μg, *P* = .1924 for 1000 μg; Student *t* test) suggest that some of the IPPD collected on the HPB PTFE Advantec filter may have been lost during the sampling process. The validation results showed that the sampling performance of the proposed method has robust reproducibility, and it also confirmed that IPPD on the HPB PTFE Advantec filter can be effectively refrigerated at 4°C for at least 7 days, indicating that this sampling method is highly likely to be usable under actual field conditions. A major limitation of this study is that the experiment used a filter spiked with IPPD standard solutions, since continuous generation of standard aerosols at known concentrations was not feasible; this is a common and crucial issue in research on sampling methods for particulate chemical substances in air. Additionally, although the validation of this method was conducted under typical laboratory conditions, which may differ from the temperature and humidity found in actual workplaces, the influence of such environmental differences on the evaluation parameters has not been investigated.

## 5. Conclusions

To our knowledge, the method described here is the first to successfully determine personal exposure to airborne IPPD within the concentration range of 0.05-4 mg/m^3^ in a 4-hour sampling period, corresponding to 1/40 to 2 times the MAK value proposed by the DFG. The developed method will be useful in assessing risks to workers handling IPPD.

## Data Availability

Data underlying this article will be made available upon reasonable request to the corresponding author.

## References

[1] PubChem . N-isopropyl-N′-phenyl-p-phenylenediamine. 2025. Accessed August 17, 2025. https://pubchem.ncbi.nlm.nih.gov/compound/7573

[2] Hartwig A, MAK Commission. N-isopropyl-N′-phenyl-p-phenylenediamine [MAK Value Documentation, 2013]. In: The MAK-Collection for Occupational Health and Safety. Deutsche Forschungsgemeinschaft, Commission for the Investigation of Health Hazards of Chemical Compounds in the Work Area; 2016:780-799.

[3] Ministry of Health, Labour and Welfare (MHLW), Japan . Guidelines for exposure assessment of workers to hazardous substances. Article in Japanese. 2020. Accessed August 17, 2025. https://www.mhlw.go.jp/content/11305000/000814711.pdf

[4] Belliardo F, Pavan I. High-pressure liquid chromatography of N-(2-propyl)-N′-phenyl-p-phenylenediamine (Ippd) and N-(1,3-dimethylbutyl)-N′-phenyl-p-phenylenediamine (Dbpd) and its application to the biomonitoring of exposed individuals. J Liq Chromatogr. 1981;4(2):279-284. 10.1080/01483918108064816

[5] Pavan I, Belliardo F, Buglione E, Massiccio MM. Biomonitoring of workers exposed to aromatic amines in rubber vulcanization. Chromatographia. 1987;24(1):651-654. 10.1007/BF02688561

[6] Pellegrino S, Petrarulo M, Testa E, Nicolotti A. Rapid high-performance liquid chromatographic determination of urinary N-(1-methylethyl)-N′-phenyl-1,4-benzenediamine in workers exposed to aromatic amines. J Chromatogr A. 1992;592(1-2):279-281. 10.1016/0021-9673(92)85096-C 1583098

[7] Ikarashi Y, Kaniwa M. Determination of p-phenylenediamine and related antioxidants in rubber boots by high performance liquid chromatography. Development of an analytical method for N-(1-methylheptyl)-N′-pheny1-p-phenylenediamine. J Health Sci. 2000;46(6):467-473. 10.1248/jhs.46.467

